# Effects of Spiritual and Religious Practices on Burnout and Academic Stress Among Undergraduate Medical Students: Protocol for a Scoping Review

**DOI:** 10.2196/88915

**Published:** 2026-10-15

**Authors:** Liahm Blank, Samir Alkhouri, Yoan Bakalov, Charlton Bassett, Hunter Triplett, Bertille Mavegam Tango Assoumou, Gary Shen

**Affiliations:** 1 Kirk Kerkorian School of Medicine at UNLV University of Nevada, Las Vegas Las Vegas, NV United States

**Keywords:** spirituality, burnout, medical students, well-being, religious coping, medical education

## Abstract

**Background:**

Burnout is increasingly recognized as a critical concern in medical education due to its associations with reduced professional performance, decreased empathy, and increased risk of medical errors. With growing evidence that medical students commonly experience burnout and its associated health consequences (eg, depression and suicide), it is essential to explore factors that may protect against its harmful effects on student well-being. Although spirituality is often considered a potential factor supporting mental health and resilience, its role in buffering burnout among medical students has not been comprehensively reviewed.

**Objective:**

This scoping review seeks to map the extent, range, and characteristics of the literature examining the relationship between spirituality and burnout and defined forms of stress among medical students. Specifically, the review will (1) describe the nature and direction of reported associations and (2) identify gaps to inform priorities for future research.

**Methods:**

This scoping review followed PRISMA-ScR (Preferred Reporting Items for Systematic Reviews and Meta-Analyses extension for Scoping Reviews) guidelines to ensure a systematic and transparent process. Studies that examined the relationship between spirituality and burnout or related outcomes and correlates, such as anxiety, depression, stress, or suicidality, among undergraduate medical students were eligible. Only studies that focused specifically on medical students rather than other health care professionals or professional students were eligible. Eligible articles were peer-reviewed and published in English. MEDLINE (PubMed), APA PsycInfo, Scopus, and Embase databases were searched. Two reviewers independently screened titles and abstracts, followed by full-text review to determine final inclusion. Discrepancies were resolved through discussion with a third reviewer until consensus was reached. Extracted data were descriptively analyzed and summarized in charts and tables and synthesized narratively according to the review questions.

**Results:**

The database searches were completed in July 2025 and yielded 1788 records. Screening was conducted from September 2025 through January 2026. Of the database records 39 (2.2%) peer-reviewed studies met the inclusion criteria. During full-text screening, articles were most frequently excluded because they did not explicitly evaluate the association between spirituality and burnout, represented an ineligible publication type, or included populations beyond undergraduate medical students without reporting separate results for medical students. Data extraction was completed in April 2026. The manuscript reporting results was completed in May 2026 and is under review as of September 2026.

**Conclusions:**

This review will provide a comprehensive overview of how spirituality and religious practices have been examined in relation to burnout and stress among undergraduate medical students. By mapping conceptualizations and experiences of spirituality within medical training, as well as varying patterns in how these practices relate to student well-being, this review highlights key gaps in the evidence and directions for future research on factors that may support medical student mental health.

**Trial Registration:**

OSF Registries osf.io/tqwsk; https://osf.io/tqwsk

**International Registered Report Identifier (IRRID):**

DERR1-10.2196/88915

## Introduction

Burnout has become a critical concern in medical education because of demanding curriculum, excessive study workloads, and the increased responsibility of caring for patients [1]. While often regarded as anticipated challenges of medical education, the academic and psychosocial stressors associated with medical school, such as sleep deprivation, peer competition, pressure to succeed, and the financial burden of student loans, contribute to significant declines in the mental health and quality of life of medical students [2]. The resulting psychological morbidity of medical students has been well described in the literature, with medical students being more likely to experience burnout compared with the general population [3]. Additionally, compared with US college graduates aged 22 to 32 years, medical students showed higher rates of emotional exhaustion, depersonalization, and overall burnout and a greater likelihood of depressive symptoms [4]. For example, a longitudinal study of 4287 medical students across 5 institutions by Dyrbye et al [5] found that burnout was reported by nearly half (49.6%) of respondents, demonstrating just how common burnout was among medical students.

As a measure of depersonalization, emotional exhaustion, and a diminished sense of personal accomplishment [6], burnout also has a well-established connection to depression and suicidal ideation [7]. Thus, burnout is a particular concern among medical students: 1 systematic review found that the overall prevalence of depression or depressive symptoms among medical students was 27.2%, and the overall prevalence of suicidal ideation was 11.1%. Across 9 longitudinal studies, included in the same systematic review that measured depressive symptoms before and during medical school, the median absolute increase in symptoms was 13.5% [8]. Depression among medical students has been further linked to substance abuse, impaired professional function, diminished physical health, and thoughts of dropping out [7,9]. Burnout can not only derail students’ careers but also harm future patients because burnout has been linked to increased errors in patient care [4,8], decreased professionalism, and an overall decline in empathy [7,10].

Medical students use a variety of strategies to cope with burnout, including religion, self-distraction, positive reframing, humor, acceptance, substance use, self-blame, and venting [11,12]. Coping mechanisms such as self-blame and substance use are associated with an increased risk of suicidal ideation, anxiety, and depression among medical students, while religion and positive reframing serve as negative predictors of these outcomes [12,13].

Several studies report spirituality as a coping strategy that medical students use to protect against burnout and its effects [12,14]. However, spirituality and religion have been defined and measured inconsistently in the literature, sometimes through single-item indicators that may not adequately capture their multidimensional nature. The National Cancer Institute defines spirituality as “having to do with deep, often religious, feelings and beliefs, including a person’s sense of peace, purpose, connection to others, and beliefs about the meaning of life” [15]. In addition, Pérez and Rohde [16] describe religion as encompassing beliefs, actions, and institutions grounded in supernatural entities, powers, or processes, whereas spirituality emphasizes belief in and the subjective experience of a relationship with God or a higher power. Therefore, religion may include more structured or institutional elements, such as rituals and community participation, while spirituality may place greater emphasis on an individual’s internal beliefs and experiences. Despite these distinctions, the constructs overlap conceptually and historically because both may involve the supernatural, transcendence, meaning, and connection to a higher power [16].

For the purposes of the present scoping review, spirituality is broadly defined to encompass organized and noninstitutional religious practices, beliefs, events, and participation associated with the ideas of connectedness and higher purpose. Wachholtz and Rogoff [17] provide an example of the protective role of spirituality on burnout, reporting that higher levels of spiritual well-being and daily spiritual experiences were associated with lower psychological distress and burnout among medical students. Similarly, Habib et al [18] found that religiosity and religious well-being were significantly and positively associated with life satisfaction.

With the potential to reduce burnout and its associated harmful effects—such as stress, anxiety, depression, and suicidal ideation—an in-depth understanding of spirituality’s protective role may improve the quality of life of medical students. While existing reviews on this topic have focused on nurses [19], physicians [20], or health care workers overall [21] or have focused separately on spirituality [22] or burnout among medical students [23], none have specifically assessed the connection between spirituality and burnout among medical students alone. This gap makes it difficult to draw medical-student-specific conclusions. Thus, a comprehensive review of current evidence on how spiritual or religious practices influence the psychosocial health of medical students is needed.

This review aims to map and synthesize the existing literature on the relationship between spirituality and burnout among undergraduate medical students worldwide. The current evidence base is highly heterogeneous in how spirituality and burnout are conceptualized, the instruments used to operationalize these constructs, and the study designs used, including cross-sectional, qualitative, and mixed methods approaches across diverse educational contexts. Given this variability, a scoping review guided by PRISMA-ScR (Preferred Reporting Items for Systematic Reviews and Meta-Analyses extension for Scoping Reviews) methodology is well suited to address our objectives, as it allows for a comprehensive examination of how key constructs are defined and measured, the range and characteristics of study populations and settings, and the nature of reported associations without aiming to estimate pooled effects. Using the population, concept, and context (PCC) framework, this review focuses on undergraduate medical students, spirituality and religious practices, and studies conducted in any geographic or educational setting. By mapping the extent and characteristics of this literature, we aim to identify conceptual and methodological gaps that may guide future research and support the development of well-grounded approaches to student well-being.

## Methods

This scoping review followed the PRISMA-ScR checklist outlined by Tricco et al [24] (Multimedia Appendix 1). In line with PRISMA-ScR guidelines and to promote methodological transparency, the protocol for this scoping review has been registered with the OSF Registries [25].

### Review Questions

Guided by the PCC framework, the review addressed the following questions:

- What associations have been reported between spirituality and positive indicators of well-being, such as happiness, life satisfaction, and stress management?
- What associations have been reported between spirituality and indicators of burnout and its consequences, including stress, anxiety, depression, and suicidality?
- What implications for medical education and medical student support can be drawn from the diversity of the explored literature?
- What gaps remain in the current literature regarding the role of spirituality in medical student burnout?

### Eligibility Criteria

#### Overview

Eligibility criteria for this scoping review were established through the PCC framework. In this review, the population consisted of undergraduate medical students; the concept was the relationship between spirituality or religious practices and burnout or related indicators of well-being; and the context included undergraduate medical education in any geographic, cultural, or educational setting.

#### Participants

The review included medical students enrolled in undergraduate medical education programs worldwide. Studies focusing on residents, fellows, physicians, nurses, or other health care professionals and trainees were excluded. In alignment with the aim of understanding spirituality’s relationship to burnout and well-being in this specific population, eligible studies were required to clearly identify medical students as the primary population of interest. Studies with the primary aim of comparing medical students and other health care trainees were excluded. Where available, demographic characteristics such as age, gender, year in training, and religious or spiritual affiliation were extracted to provide contextual insights into the populations studied.

#### Concept

This review examined studies that investigated how spirituality, encompassing personal spiritual beliefs, religious practices, and overall spiritual well-being, related to burnout and its negative consequences. These harmful effects included stress, emotional exhaustion, and broader measures of mental distress, such as anxiety, depression, pessimism, and suicidality. Studies were required to explicitly assess the interaction between spirituality and burnout. Those that explored only one of these concepts without linking them were excluded. Eligible studies were required to include a substantive focus on spirituality as either a variable of interest, a component of an intervention, or a subject of reflection. Studies in which spirituality was only mentioned tangentially (eg, references to “team spirit” or general morale) were excluded. Both protective and adverse associations between spirituality and well-being outcomes were included.

#### Context

This review considered studies conducted among medical students worldwide. Research conducted exclusively in hospitals to examine spirituality and burnout among health care professionals or patients was excluded. While eligible studies were required to be conducted among medical school populations, measures of spiritual belief were not limited to the context of medical education.

#### Types of Sources

This scoping review included peer-reviewed, full-text articles published in English through February 1, 2025. Eligible study designs included qualitative studies, randomized controlled trials, nonrandomized controlled trials, prospective and retrospective cohort studies, case-control studies, cross-sectional studies, and mixed methods studies that met the inclusion criteria. This review excluded conference abstracts and proceedings, dissertations and theses, opinion pieces, editorials, letters to the editor, book chapters, book reviews, newsletters, narrative and systematic reviews, and other non–peer-reviewed or gray literature.

### Information Source

#### Preliminary Search for Planned or Ongoing Studies

A preliminary search of PubMed, APA PsycInfo, Embase, and Scopus databases was conducted on March 3, 2025, to identify any published or ongoing reviews on the topic. In addition, a search of PROSPERO was conducted on May 30, 2025. These searches found no published or ongoing scoping or systematic reviews on the topic.

#### Search Strategy

The search strategy was developed by the research team in collaboration with the undergraduate medical librarian. A preliminary search of PubMed was undertaken to identify articles on the topic and extract keywords from the title and abstract of relevant articles. The search used the PCC framework to address the review questions through three concept blocks: (1) undergraduate medical students; (2) spirituality, religiosity, and related practices or coping; and (3) burnout and indicators of well-being. Controlled vocabulary and free-text synonyms within each concept block were combined using the Boolean operator OR, and the 3 concept blocks were combined using AND. Quotation marks were used for multiword phrases, and truncation symbols were used to retrieve word variants where supported by the database. The search strategy was adapted to the indexing terms and phrase-searching rules of each database. Final searches were conducted in MEDLINE via PubMed, APA PsycInfo, Scopus, and Embase databases in July 2025. MEDLINE and Embase databases were selected for their coverage of biomedical and health sciences research, APA PsycInfo database for psychological and behavioral research, and Scopus database for its broad multidisciplinary and citation-indexing coverage. Web of Science database was not searched because Scopus was selected as the multidisciplinary citation database to complement the 3 subject-specific databases, considering the substantial overlap in journal coverage between Scopus and Web of Science [26].

Searches were limited to English-language publications published through February 1, 2025. The restriction to English-language publications was based on the language expertise of the research team and the absence of resources for the reliable translation and assessment of non–English-language full-text articles. No publication type or study design filters were applied during the database searches; these criteria were assessed during screening. In addition, the reference lists of all included articles were screened for additional papers. Gray literature was not searched, and study authors were not contacted for additional information. The complete search strategy for each database is provided in Table 1.

**Table 1:** Search strategy.

Database	Search string	Records retrieved
MEDLINE (PubMed)	(“Burnout, Psychological” [Mesh] OR “Stress, Psychological” [Mesh] OR burnout[tiab] OR “burn out” [Tiab] OR stress[tiab] OR fatigue[tiab] OR exhaustion[tiab] OR well-being[tiab]) AND (“Religion and Psychology” [Mesh] OR spiritual*[tiab] OR religio*[tiab] OR pray*[tiab] OR coping[tiab]) AND (“Students, Medical” [Mesh] OR “Education, Medical, Undergraduate” [Mesh] OR “medical student” [Tiab])	492
APA PsycInfo	(Burnout OR burn out OR exhaustion OR fatigue OR stress OR well-being) AND (spiritual* OR religio* OR pray* OR coping) AND “medical student”	254
Scopus	(Burnout OR “burn out” OR exhaustion OR fatigue OR stress OR well-being) AND (spiritual* OR religio* OR pray* OR coping) AND “medical student”	1105
Embase	(“Burnout”/exp OR “mental stress”/exp OR “mental fatigue”/exp OR “exhaustion”/exp OR “well-being”/exp OR “burn out”:ab,ti OR “well-being”:ab,ti OR “exhaustion”:ab,ti OR “fatigue”:ab,ti OR “stress”:ab,ti) AND (“religion”/exp OR “meditation”/exp OR “yoga”/exp OR “coping”/exp OR religio*:ab,ti OR pray*:ab,ti) AND (“medical student”/exp OR “medical student”:ab,ti)	891

### Study Selection

After executing the search in the 4 databases, all initially selected articles were uploaded to Rayyan. Using Rayyan, all duplicate articles and review articles were removed. Next, titles and abstracts were screened by 2 independent reviewers, adhering to the specified inclusion and exclusion criteria; any discrepancies in inclusion or exclusion decisions were resolved through discussion with a third reviewer until consensus was reached. Full texts of articles included based on their titles and abstracts were then reviewed to determine their eligibility. Discrepancies in inclusion or exclusion decisions between the initial 2 independent reviewers were resolved by a third reviewer.

### Quality Appraisal

In line with the methodological guidance outlined in the PRISMA-ScR checklist, this scoping review does not include a formal appraisal of the methodological quality or risk of bias of the included studies. Excluding quality appraisal enables the inclusion of a wide range of study designs and evidence types, thus providing a comprehensive overview of the topic.

### Data Extraction

Data were independently extracted from all full-text articles included in the scoping review by 3 independent reviewers. The specific data items collected are outlined in Table 2. An additional reviewer resolved conflicting decisions between reviewers.

**Table 2:** Data extraction instrument.

Main category	Subcategory
Article identification	Title Year of publication DOI Full citation
Research purpose	What measure(s) related to well-being is/are being evaluated? What measure(s) related to burnout is/are being evaluated? What measure(s) related to spirituality is/are being evaluated? What is the stated research question?
Study population	What is the sample size? What is the study’s geographic focus or country of publication? Which year of medical school were the students being evaluated in? Were any exclusion criteria identified?
Study design/methods	Was the data qualitative, quantitative, or mixed? What was the study design? Sampling method: How were participants recruited? What scales, if any, were used to assess spirituality? What scales, if any, were used to assess burnout/well-being and its related components/factors?
Major findings	What was the main focus of the analysis identified by the authors? What demographic data were presented in the analysis? What conclusions were made about spirituality in relation to well-being? What conclusions were made about spirituality in relation to burnout? What limitations were noted by the authors? What was the main conclusion identified by the authors? Were future research opportunities identified?

### Data Analysis and Presentation

Extracted data from the included studies were summarized and presented using charts and tables organized according to the review objectives and reflected the established inclusion criteria. Study characteristics, including publication year, country or region, study design, sample size, and year of medical training, were summarized to describe the extent and distribution of the evidence. Definitions and measures of spirituality, religiosity, burnout, and related well-being outcomes were compared to identify conceptual and measurement patterns. Reported relationships were grouped into three outcome domains: (1) burnout and its dimensions, including emotional exhaustion, cynicism, and professional efficacy; (2) stress and related adverse mental health outcomes, including anxiety, depression, psychological distress, and suicidality; and (3) positive indicators of well-being, including resilience, empathy, happiness, life satisfaction, and quality of life. Within each domain, findings were categorized as protective or inverse, adverse or positive, null, or mixed.

Qualitative and mixed methods findings were summarized narratively within the same outcome domains to preserve contextual information. Reported implications for medical education and student support, study limitations, and recommendations for future research were also summarized to identify evidence gaps and research priorities.

### Ethical Considerations

Ethics approval was not required as this scoping review used previously published data from existing literature. We plan to publish the results in a peer-reviewed open-access journal, and its findings may also be presented at relevant mental health and medical education conferences. Findings from this review may inform educators, medical students, and institutions on the potential role of spirituality in addressing burnout and guide the development of evidence-based interventions promoting mental health in medical training.

## Results

Formal data collection began when the final database searches were completed in July 2025. These searches yielded 1788 records, of which 39 peer-reviewed studies met the inclusion criteria. During full-text screening, articles were most frequently excluded because they did not explicitly evaluate the association between spirituality and burnout, represented an ineligible publication type, or included populations beyond undergraduate medical students without reporting separate results for medical students. The complete study selection process and all reasons for exclusion are presented in Figure 1.

**Figure 1:**
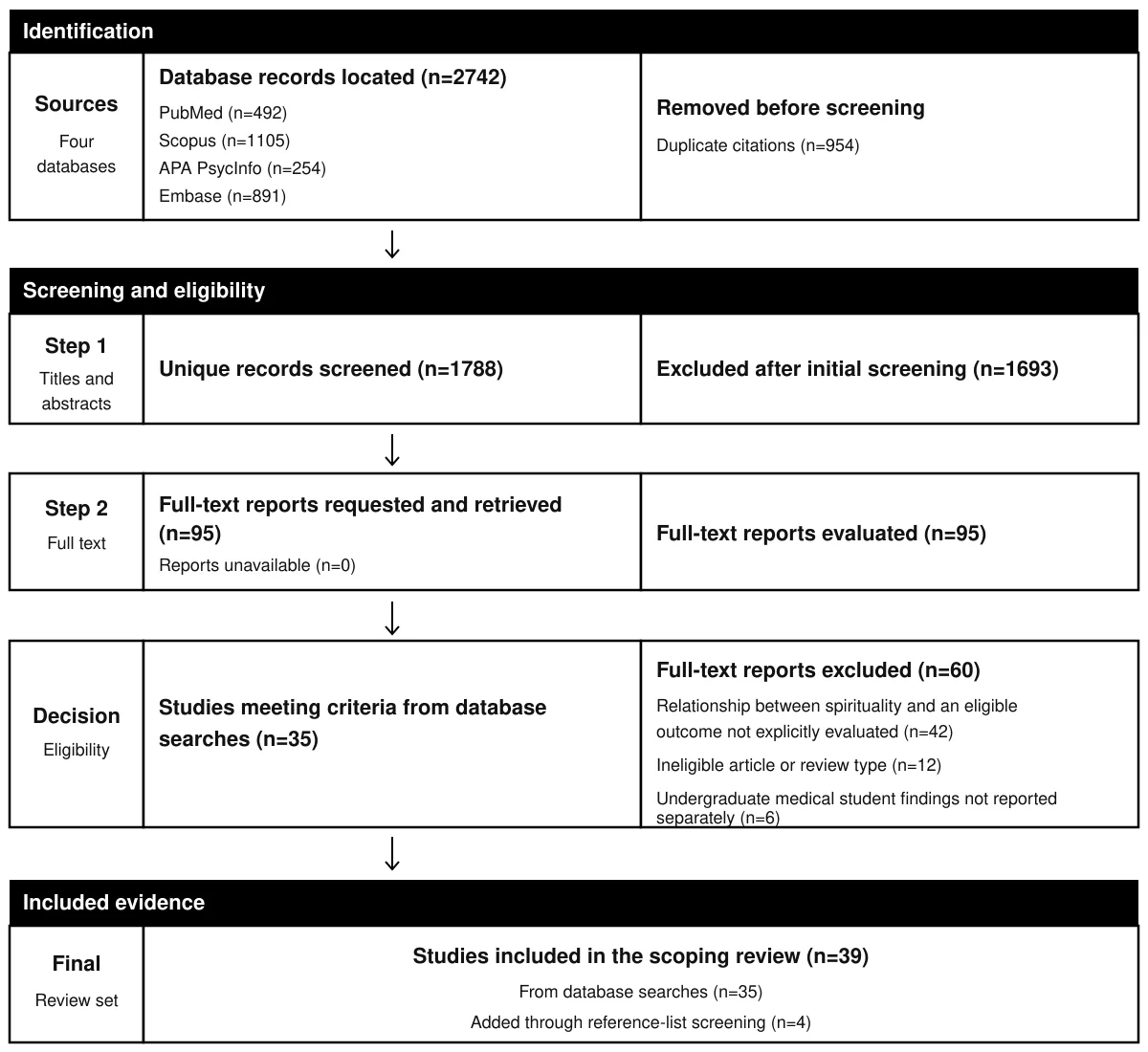
Flow of records through identification, screening, eligibility assessment, and inclusion.

Data extraction was conducted from March through April 2026. The included literature spanned publications from 2007 through 2025 and represented 19 countries across 5 continents. The studies varied in their definitions and measurement of spirituality, religiosity, burnout, psychological distress, and positive well-being. Most used observational designs, while a smaller number used longitudinal, qualitative, or mixed methods approaches. These variations supported the decision to map and narratively synthesize the evidence rather than conduct a quantitative meta-analysis.

The review has been completed, and the manuscript reporting results was completed in May 2026 and is under review as of September 2026. The review was completed without deviations from the registered protocol.

## Discussion

### Principal Considerations

Existing reviews have examined spirituality and well-being among physicians, nurses, and health care professionals more broadly [19-21], while others have addressed either spirituality or burnout among medical students without focusing specifically on the relationship between these constructs [22,23]. De Diego-Cordero et al [19] reported that spirituality and religiosity were correlated with lower levels of burnout, depersonalization, and exhaustion among nurses but emphasized the limited number of experimental studies evaluating whether spiritually oriented interventions can reduce burnout. Similarly, Whitehead et al [20] concluded that burnout among physicians may be associated with spiritual health, although the evidence was less consistent for religion and remained limited by methodological weaknesses. A broader review of health care professionals also identified spiritual well-being as a potentially relevant correlate of burnout while noting substantial variation in how these constructs were defined and measured [21].

Individual studies among medical students reported inverse associations between spirituality and dimensions of burnout or psychological distress [5,15]. However, these studies did not provide a comprehensive account of the geographic, conceptual, and methodological characteristics of the broader evidence. Related reviews have generally addressed different questions. For example, Wenham et al [22] focused on spirituality within medical education curricula, including the knowledge and skills needed to provide spiritual care, whereas Nowińska et al [23] reviewed burnout among medical students without specifically examining its relationship with spirituality or religious practices.

A medical student–specific synthesis of how spirituality and related concepts have been defined, measured, and examined in relation to burnout and other indicators of mental health and well-being is lacking. This scoping review was designed to address this gap by mapping the scope and characteristics of the available evidence. Its broad approach allowed consideration of spirituality, religiosity, religious coping, and spiritual well-being alongside burnout, psychological distress, and positive dimensions of well-being. It also permitted examination of diverse study designs and cultural contexts, which was important because the meaning and expression of spirituality may differ across populations and educational environments.

### Implications for Research and Practice

By documenting the concepts, measures, populations, and study designs represented in the literature, this review may help clarify how research on spirituality and medical student well-being has developed and where important evidence gaps remain. The review may also support greater conceptual precision by encouraging researchers to distinguish among spirituality, religiosity, religious practices, and religious coping rather than treating these constructs as interchangeable. These insights may help guide the development of more rigorous, context-sensitive studies aimed at understanding how spirituality may relate to well-being during medical training. In practice, the review may also support educators and program leaders in considering broader dimensions of student well-being, including personal or existential resources that have received comparatively little attention in the literature. Potential applications may include accommodations that support students’ religious or spiritual practices, optional and inclusive opportunities for reflection, and access to spiritual care or chaplaincy services.

### Strengths and Potential Limitations

This scoping review protocol follows PRISMA-ScR guidelines and incorporates a clearly defined, multistage screening process involving independent reviewers. The search strategy was developed in collaboration with a medical librarian and tailored to 4 major databases (PubMed, Embase, APA PsycInfo, and Scopus) using both keyword and index-term approaches, enhancing the breadth, rigor, and reproducibility of the search. However, the review only included peer-reviewed articles from established databases, excluding gray literature, which may limit the comprehensiveness of the evidence captured. Additionally, restricting inclusion to English-language publications may introduce language bias and reduce the global applicability of the findings. In addition, consistent with the purpose of a scoping review, the methodological quality and risk of bias of the included studies were not formally appraised. The review therefore characterizes the extent and nature of the available evidence but cannot establish causal relationships or determine the effectiveness of spirituality-related interventions.

## Data Availability

All data produced and analyzed for this review will be included in the final publication and will be available from the corresponding author on reasonable request.

## Appendix

Multimedia Appendix 1 PRISMA-ScR checklist.

## Abbreviations

PCC: population, concept, and context
PRISMA-ScR: Preferred Reporting Items for Systematic Reviews and Meta-Analyses extension for Scoping Reviews
